# Cucurbitacin B exerts anti-cancer activities in human multiple myeloma cells *in vitro* and *in vivo* by modulating multiple cellular pathways

**DOI:** 10.18632/oncotarget.10584

**Published:** 2016-07-13

**Authors:** Tai Yang, Jin Liu, Mali Yang, Ning Huang, Yueling Zhong, Ting Zeng, Rong Wei, Zhongjun Wu, Cui Xiao, Xiaohua Cao, Minhui Li, Limei Li, Bin Han, Xiaoping Yu, Hua Li, Qiang Zou

**Affiliations:** ^1^ School of Pharmacy, Chengdu Medical College, Chengdu, China; ^2^ Department of Immunology, Chengdu Medical College, Chengdu, China; ^3^ Laboratory for Aging Research, State Key Laboratory of Biotherapy and Cancer Center, West China Hospital, Sichuan University and Collaborative Innovation Center for Biotherapy, Chengdu, China; ^4^ Department of Public Health, Chengdu Medical College, Chengdu, China; ^5^ Cancer Center, Chengdu Military General Hospital, Chengdu, China

**Keywords:** cucurbitacin B, multiple myeloma, Aurora A kinase, multiple cellular pathways, xenograft

## Abstract

Cucurbitacin B (CuB), a triterpenoid compound isolated from the stems of *Cucumis melo*, has long been used to treat hepatitis and hepatoma in China. Although its remarkable anti-cancer activities have been reported, the mechanism by which it achieves this therapeutic activity remains unclear. This study was designed to investigate the molecular mechanisms by which CuB inhibits cancer cell proliferation. Our results indicate that CuB is a novel inhibitor of Aurora A in multiple myeloma (MM) cells, arresting cells in the G2/M phase. CuB also inhibited IL-10-induced *STAT3* phosphorylation, synergistically increasing the anti-tumor activity of Adriamycin *in vitro*. CuB induced dephosphorylation of cofilin, resulting in the loss of mitochondrial membrane potential, release of cytochrome c, and activation of caspase-8. CuB inhibited MM tumor growth in a murine MM model, without host toxicity. In conclusion, these results indicate that CuB interferes with multiple cellular pathways in MM cells. CuB thus represents a promising therapeutic tool for the treatment of MM.

## INTRODUCTION

Multiple myeloma (MM) is a malignant clonal plasma disorder characterized by damage to multiple organs including bone lesions, hypocalcemia, anemia, and renal insufficiency [1]. MM accounts for 2% of all malignancies and is the second leading hematological malignancy [2]. Conventional therapies for MM include chemotherapy and radiation therapy, and although, several chemotherapeutic agents, such as adriamycin, lenalidomide, thalidomide, and bortezomib have improved outcomes of MM patients, this disease remains incurable with a 5-year survival rate between 5% and 19% [3]. Therefore, novel, safe, and effective agents are urgently required to treat MM.

**Figure 1:**
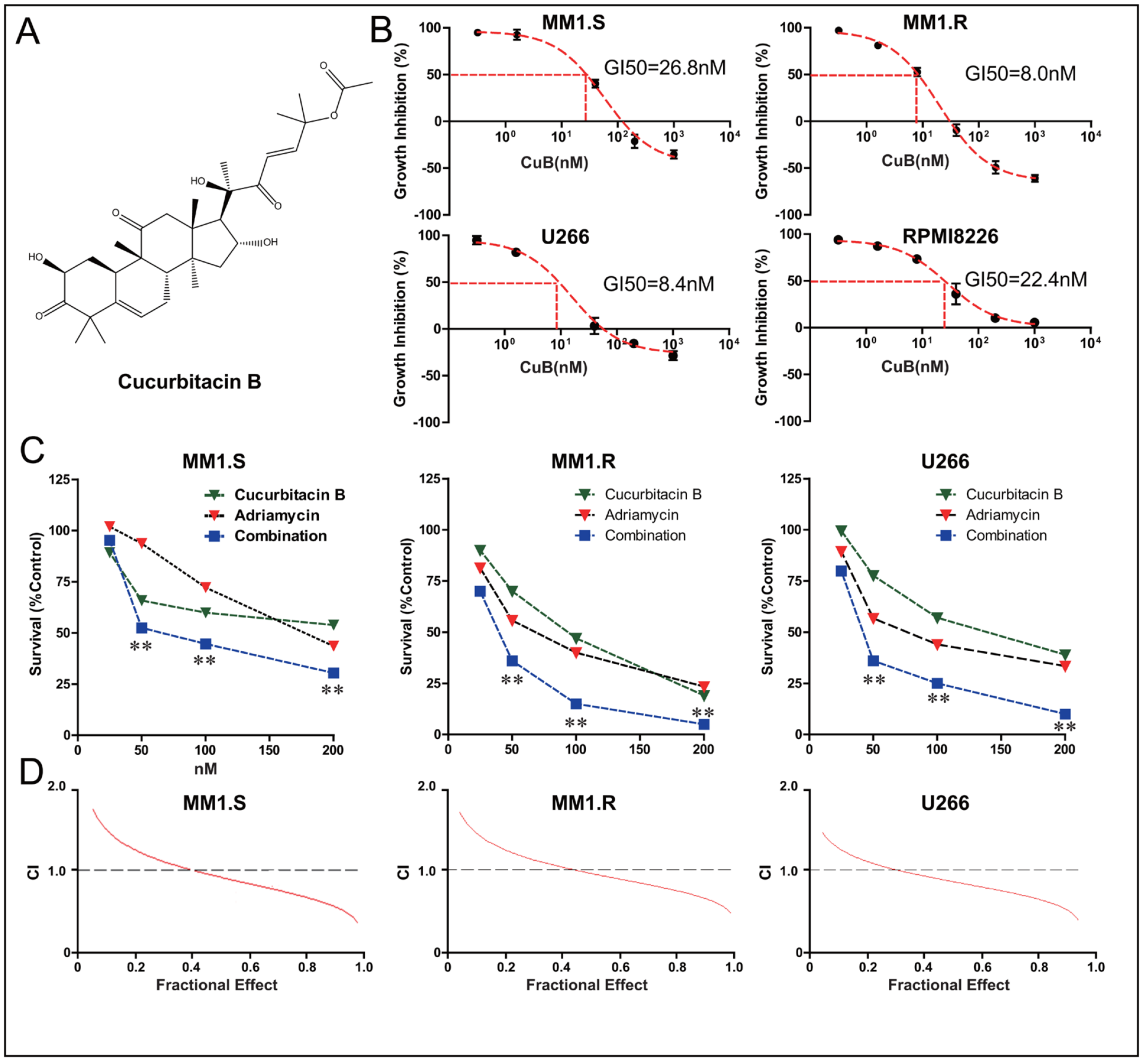
CuB inhibits proliferation of MM cells, acting synergistically with Adriamycin **A**. Chemical structure of CuB. **B**. CuB reduced MM cell viability in a dose-dependent manner, with an IC50 ranging from 8 to 26.8 nM. **C**. Combination of CuB and Adriamycin synergistically reduced growth of MM cells. The fraction of surviving cells in each group was assessed by CCK-8 assay. Presented data are representative of three independent experiments. Statistical significance of differences was assessed by the Student *t*-test. ** *P* < 0.01. **D**. CuB and the Adriamycin exerted a synergistic effect on growth inhibition in MM cells. A CCK-8 assay was employed and isobologram analysis was used to determine the mode of the effects of CuB and Adriamycin combinations at equitoxic concentrations in the MM1.S, MM1.R, and U266 cells. CI, combination index, was calculated using Calcusyn software, and CI < 1.0 corresponded to a synergistic interaction.

In our efforts to identify novel anticancer agents, special attention has been paid to natural products. Natural products and derivatives represent the majority of currently used chemotherapeutic agents in the clinic and in the pipeline of new cancer drugs under development [4]. Cucurbitacins B (CuB, Figure 1A) and E, have been isolated from from Tian Gua Di (Cucumis melo fruit peduncle), a traditional Chinese medicine that has been shown to effectivly treat liver diseases such as chronic hepatitis and liver malignancies [5]. More recently, CuB and related compounds have been shown to exert anticancer activity in *in vitro* and *in vivo* models, of hepatoma [6], colorectal cancer [7], breast cancer [8], neuroblastoma [9], myeloid leukemia [10], pancreatic cancer [11], lung cancer [12], and melanoma [13]. These promising results prompted us to evaluate the possibility of using CuB to treat MM, either alone or in combination with current chemotherapeutic agents, to improve response rates and reduce toxicity.

One major hurdle to the development of natural product-based anticancer agents is identifying their molecular target(s) and defining their underlying mechanism(s) of action. Although the antitumor activity of CuB has been intensively investigated, its mechanism of action remains controversial. Its anti-proliferative effects have been associated with cell cycle arrest and apoptosis, mediated via inhibition of *STAT3* signaling [14, 15], but some reports argue that its antitumor activity is independent of effects on the *STAT3* pathway [16, 17], and while blocking *STAT3* signaling typically induces G0/G1 arrest [18, 19], CuB and its analogs induce G2/M arrest [9, 20], and direct interaction of CuB and STAT3 has not been demonstrated. Clarifying the role of *STAT3* and other kinases in CuB's anticancer activity may not only further its development as novel anticancer agent but also elucidate the role of *STAT3* in cancer therapy. Kinases have been one of the hottest classes of molecular targets for cancer drug discovery and development. Advances in high-throughput screening technology, with a range of surface chemistry and activation strategies, have provided a powerful tool for evaluation of chemical-protein interactions and kinase activity inhibition, target identification, and signal pathway elucidation [21]. In this study we employed kinase screening approaches to identify kinase targets of CuB, and sought to identify the molecular mechanisms responsible for CuB-induced apoptosis.

CuB treatment was reported to induce de-phosphorylation of Cofilin, a key regulator of actin filament dynamics, causing cell cycle arrest and apoptosis [10, 16]. Dephosphorylated cofilin can be translocated into mitochondria, disturbing mitochondria function or enhancing translocation of pro-apoptotic proteins in the mitochondria. Thus altering mitochondrial membrane potential, triggering release of cytochrome c (Cyt c), and apoptosis [22, 23]. Here we attempt to define the role of dephosphorylation of cofilin in the anticancer activity of CuB.

One characteristic effect of aurora kinase inhibition is cell cycle arrest in the G2/M phase [24, 25]. In this study we also sought to demonstrate that CuB could act as a novel Aurora A inhibitor in induced MM cells, arresting cells in the G2/M phase. Considering that IL-10 could enhance proliferation of MM cells, and reduce Adriamycin-induced cell death, we hypothesized that CuB-mediated inhibition of the *STAT3* pathway might synergistically enhance the anti-tumor activity of Adriamycin. Additionally, we sought to investigate the relationship between CuB-induced cofilin dephosphorylation and mitochondrial dysfunction. Through these experiments, we aimed to elucidate the mechanism by which CuB reduces proliferation of MM cells, and to provide a basis for the development of this compound as a potential therapeutic agent for the treatment of MM.

## RESULTS

### CuB, administered alone or in combination with Adriamycin, inhibits MM proliferation

Proliferation of dexamethasone-resistant (MM1.R) and dexamethasone-sensitive (MM1.S), and U266, and RPMI8226 cells incubated with CuB for 24 h was significantly inhibited in a dose-dependent manner. Interestingly, MM1.R cells were more sensitive to CuB than MM1.S cells (Figure 1B).

Furthermore, in order to investigate synergy of CuB and Adriamycin, cells were incubated with both CuB (0, 25, 50, 100 and 200 nM) and Adriamycin (0, 25, 50, 100 and 200 nM) in a checkerboard fashion. Cell viability was assessed after 72 h. Combination treatment inhibited proliferation more effectively than either agent alone (Figure 1C). Proliferation of MM1.S, MM1.R and U266 cells was substantially inhibited in the presence of 50, 100 and 200 nM CuB and Adriamycin, while 50 nM Adriamycin alone did not exert significantly anti-proliferative activity. The combination of CuB with Adriamycin exhibited a synergistic effect (CI < 1) at IC_50_s (fraction of cells affected = 0.5) in MM1.S cells (Figure 1D).

### CuB induces apoptosis in MM cells

**Figure 2:**
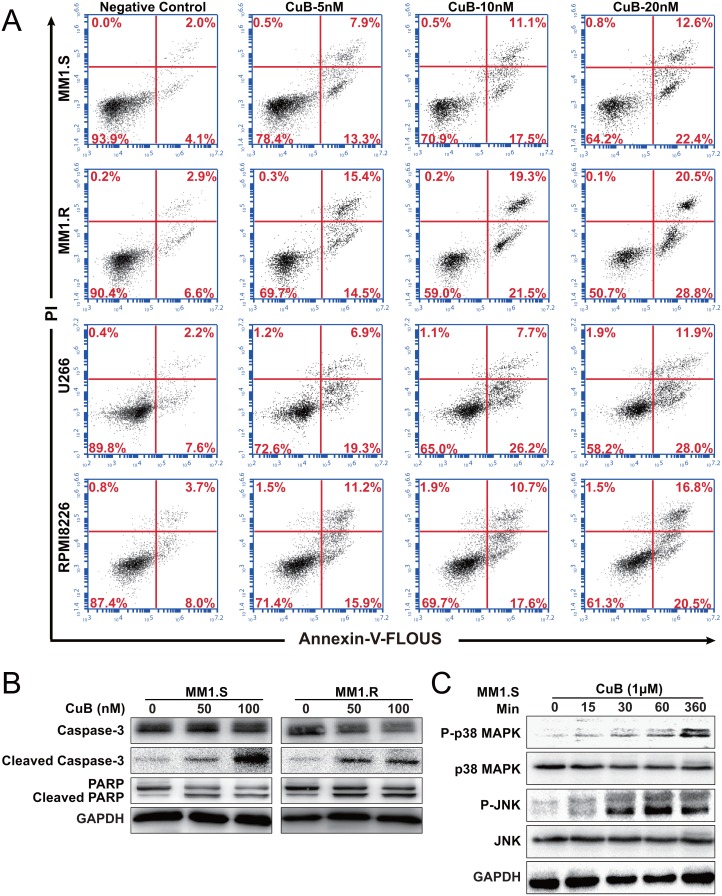
CuB induces apoptosis in MM cells **A**. MM1.S, MM1.R, U266 and RPMI8226 cells apoptosis was assessed using dual staining with Annexin V and PI. 20 nM CuB increased the fraction of apoptotic cells from 4.1% to 22.4% in MM1.S cells, 6.6% to 28.8% in MM1.R cells, 7.6% to 28.0% in U266 cells and 8.0% to 20.5% in RPMI8226 cells. **B**. Effects of CuB on the levels of caspase-3, PARP and their cleavage forms in the MM1.S and MM1.R cells by Western Blot. **C**. Effects of CuB on the levels of phospho-p38 MAPK, phospho-JNK, p38 MAPK, and JNK in the MM1.S and MM1.R cells by Western Blot.

To confirm whether CuB caused apoptosis, the percentage of Annexin V-positive cells was measured using flow cytometry. CuB increased the fraction of cells undergoing early apoptosis (annexin V positive, PI negative) in a dose-dependent manner (Figure 2A). Addition of 20 nM CuB for 48 h increased the fraction of MM1.S cells undergoing apoptosis from to 4.1% to 22.4%, the fraction of MM1.R cells undergoing apoptosis from 6.6% to 28.8%, the fraction of U266 cells undergoing apoptosis from 7.6% to 28.0%, and the fraction of RPMI8226 cells undergoing apoptosis from 8.0% to 20.5%.

In order to assess the contributions of active caspases to CuB-induced apoptosis, cleavage caspase-3 and PARP levels were measured in MM cells following CuB treatment (Figure 2B). CuB indeed induced apoptosis of MM cells, consistent with the results of the growth-inhibition assay.

Many studies have reported an important role of the mitogen-activated protein kinase (MAPK) in apoptosis signaling and interactions between MAPK and caspase pathways may determine the fate of a cell in response to various stimuli [26]. Therefore, CuB-induced activation of MAPKs was evaluated using Western blot analysis. As shown in Figure 2C, 1 μM CuB induced significant JNK activation without any change in total JNK protein levels. Increased p38 activation was also time-dependent, peaking at 6 h. p38 MAPK was previously reported to be a critical regulator of G2-M arrest [27], which was consistent with the results of CuB-induced G2-M cell cycle arrest in MM cells. These findings suggest that CuB-induced apoptosis involved p38 MAPK activation.

### CuB suppresses Aurora A activity

**Figure 3:**
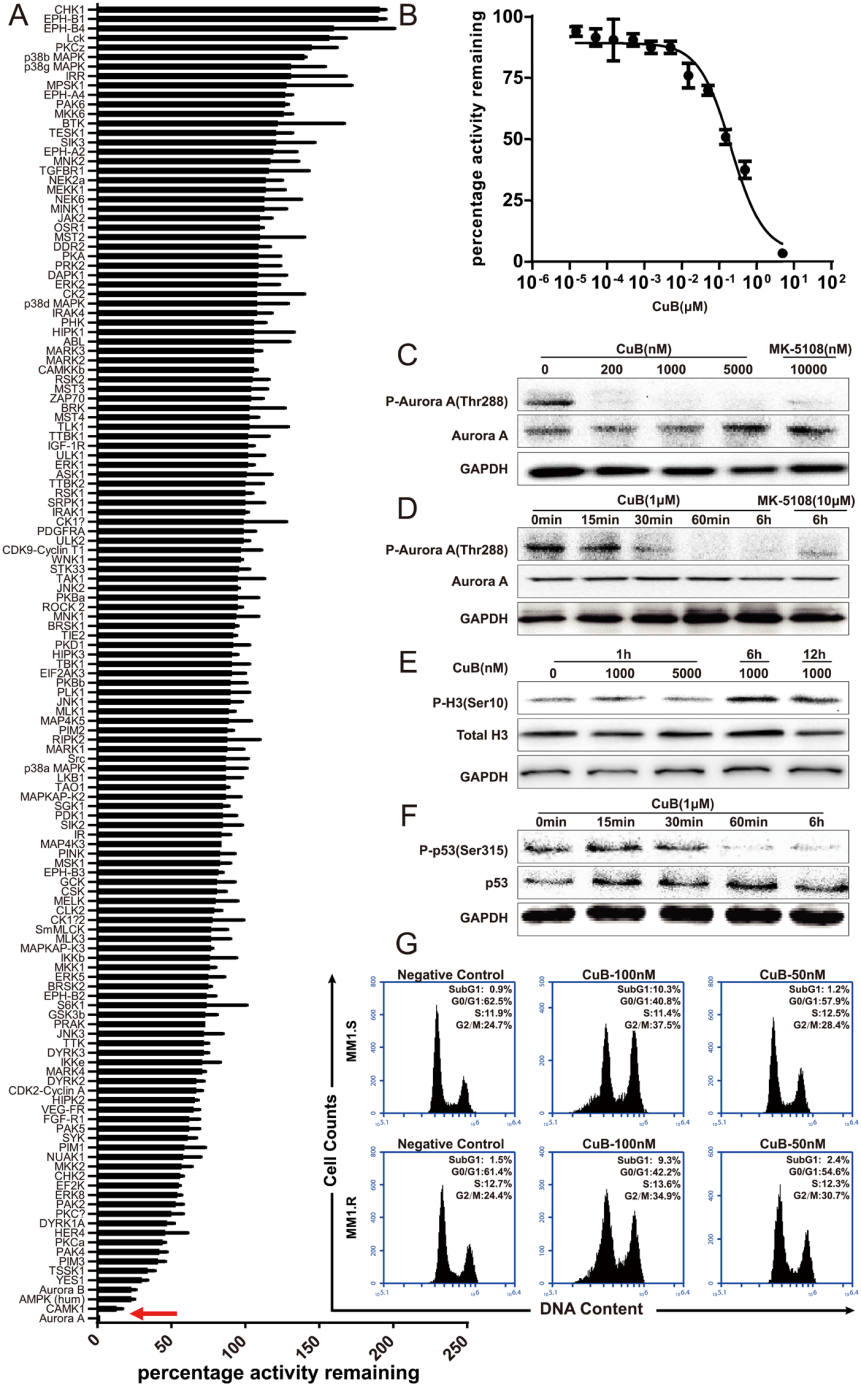
CuB is a novel potent Aurora A kinase inhibitor **A**. The inhibitory activity of CuB was assessed against a panel of 140 protein kinases. Results presented indicate the remaining activity of each kinase (average of two duplicate determinations) in the presence of 500 nM of CuB ± standard deviation. Results are ranked according to the percentage of the remaining Aurora A treated with 500 nM CuB. Inhibition of Aurora A kinase by CuB is indicated by a red arrow. **B**. Aurora A kinase activity in the presence or absence of increasing concentrations of CuB. The results are presented as the percentage of kinase activity relative to the control (DMSO) and the average of at least duplicate reactions where similar results were obtained. **C**. CuB inhibited phosphorylation of Aurora A. **D**. CuB inhibited phosphorylation of Aurora A in a time-dependent manner. **E**. Histone H3 phosphorylation at S10 was analyzed by Western blotting. **F**. CuB suppressed phosphorylation of p53 in a time-dependent manner. **G**. Cell cycle profiles of propidium iodide-stained cells after treatment with CuB (0, 0.1 and 0.05 μM) for 24 h show a G2/M arrest.

The kinase selectivity of CuB was assessed using a radioactivity-base enzymatic assay against a kinases panel (Dundee profiling) [28]. CuB inhibited the kinase activity of p-Aurora A by over 90% at 500 nM (Figure 3A), with an IC_50_ value of 100 nM (Figure 3B), indicating that CuB is a potent inhibitor of Aurora A.

The effect of CuB on Aurora A autophosphorylation at Thr-288 (pT288) was assessed in MM1.S cells using Western blotting. As shown in Figure 3C. 200, 1000 and 5000 nM CuB for 1 h inhibited Aurora A auto-phosphorylation of T288, but did not affect the total Aurora A content of cells, suggesting that the decreased phosphorylation of T288 was a result of reduced Aurora A phosphorylation rather than degradation or down-regulation of Aurora A protein. The inhibitory effect was also shown to be time-dependent (Figure 3D).

In order to assess the selectivity of CuB for specific inhibition of Aurora A kinase over the structurally related Aurora B kinase, Western blotting analysis was performed, and phosphorylation of Histone H3 on Ser10, an Aurora B-specific substrate in cells, was quantified. Although 1000 and 5000 nM CuB suppressed Aurora A activity, these concentrations of CuB were not sufficient to inhibit Aurora B activity, as demonstrated by the phosphorylated Histone H3-immunopositive reaction in the MM1.S cells (Figure 3E). Furthermore, treatment of MM1.S cells with CuB increased phosphorylation of histone H3; the latter is the marker of mitotic cells and can be modulated by Aurora B [29], indicating that CuB only inhibited Aurora A phosphorylation, not Aurora B. As a substrate of Aurora A kinase, p53 can be phosphorylated at Ser315 [30], and therefore, the effects of CuB on phosphorylation of p53 at Ser315 in MM1.S cells were assessed. As expected, CuB reduced phosphorylation of p53, further confirming the specificity of CuB for Aurora A kinase (Figure 3F). Taken together, these results indicate that CuB suppresses activation of Aurora A, without inhibiting Aurora B.

Considering that a characteristic effect of Aurora kinase inhibition is cell cycle arrest in the G2/M phase [24, 25], the effect of CuB on the cell cycle in MM cells was examined. Treatment with 50 and 100 nM CuB for 48 h induced significant G2/M cell cycle arrest in MM1.S cells, in a dose-dependent manner. Incubation with 100 nM CuB increased proportion of cells with 4N DNA content from 24.7% to 37.5% in MM1S cells, and from 24.4% to 34.9% in MM1.R cells (Figure 3G).

### CuB inhibits IL-10-mediated *STAT3* phosphorylation in RPMI8226 cells

**Figure 4:**
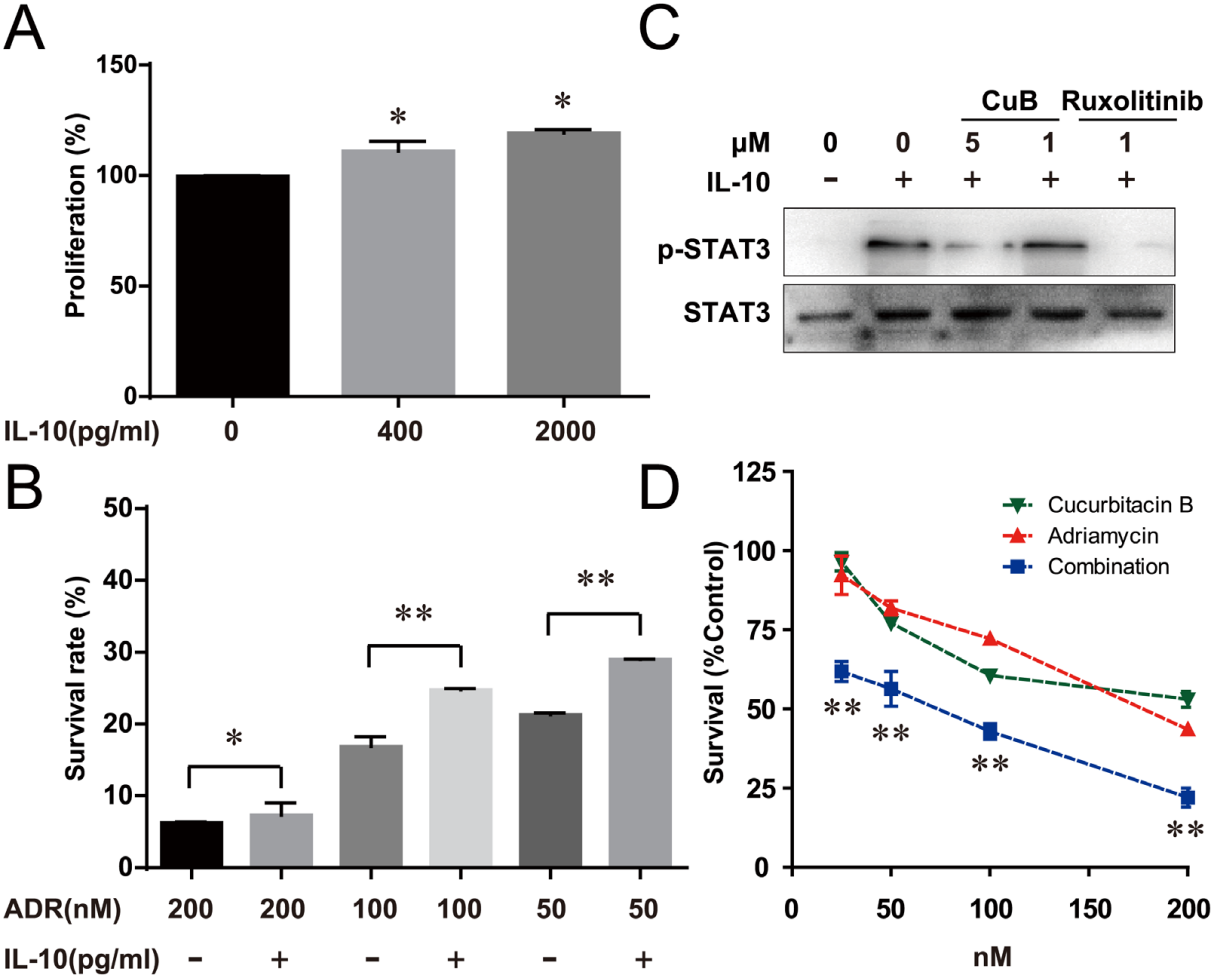
CuB Inhibition of the IL-10/STAT3 pathway significantly increased the cell sensitivity to Adriamycin **A**. IL-10 increased proliferation of RPMI8226 cells by about 120%. **B**. RPMI8226 cells were pretreated with the indicated doses of Adriamycin, followed by 2000 pg/ml IL-10 for 72 h. Proliferation was assessed by CCK-8 assay. **C**. Serum-starved RPMI8226 cells were pretreated with the indicated doses of CuB for 30 min, then incubated with 50 ng/ml IL-10 for 10 min. At the indicated times, the cellular proteins were extracted and analyzed by Western blotting using antibodies against *STAT3* and phospho-*STAT3*. **D**. Cells were treated with the indicated doses of Adriamycin alone or in combination with CuB for 72 h, and proliferation was assessed by CCK-8 assay. Statistical significance was assessed by the Student *t*-test, ** *P* < 0.01,* *P* < 0.05.

CuB was previously reported to inhibit activation of *STAT3* in cancer cells [14, 31], so we evaluated the inhibitory effect of CuB on *STAT3* in MM cells. RPMI8226 cells were serum starved for 6 h, and pretreated with various concentrations of CuB before stimulation with IL-10 (50 ng/mL). The treatment was terminated by centrifugation at 300 g for 5 min and cells were washed twice with PBS and lysed at 4°C in lysis buffer. Western blotting indicated that 5 μM CuB for 10 min inhibited IL10-induced *STAT3* activation while 1 μM CuB has no effect (Figure 4C). To further understand the importance of the IL-10/*STAT3* pathway in chemoresistance, survival, and proliferation of multiple myeloma, IL-10-enhanced proliferation was assessed using a CCK-8 assay. Figure 4A shows the proliferative effects of IL-10. IL-10 increased RPMI8226 proliferation by 120%. This finding was consistent with previous studies that have demonstrated IL-10 enhances proliferation of MM cells [32]. Further, IL-10 inhibited Adriamycin-induced cell death of RPMI8226 cells (Figure 4B). Further, we determined whether CuB-induced inhibition of IL-10/*STAT3* could enhance Adriamycin's anti-proliferative effect in RPMI8226 cells. The combination of CuB and Adriamycin exhibited a synergistic inhibitory effect on RPMI8226 cell growth (Figure 4D), further suggesting that activation of *STAT3* plays an important role in MM, and providing a rationale for combination therapy using a *STAT3* inhibitor and other chemotherapeutic drugs.

### CuB induces inhibitory phosphorylation of cofilin in MM cells

**Figure 5:**
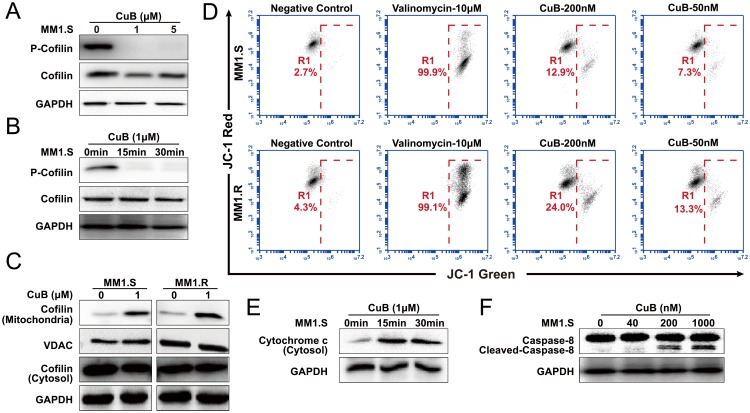
CuB induces dephosphorylation of cofilin, which is translocated to mitochondria, causing mitochondrial disfunction, release of cytochrome c and activation of caspase-8 **A**. MM1.S cells were pretreated with various concentrations of CuB (1 and 5 μM), cell lysates were analyzed by Western blot, using anti-phospho-cofilin antibodies, and both 1 and 5 μM CuB induced dephosphorylation of cofilin. **B**. MM1.S cells were pretreated with 1 μM of CuB at the indicated time intervals. Total cellular extracts were prepared and subjected to Western blot analysis using antibodies against phospho-cofilin. **C**. Cytosolic and mitochondrial fractions of control and 1 μM CuB-treated cells for 1 h were prepared and subjected to immunoprecipitation using an anti-cofilin antibody and anti-VDAC antibody, followed by immunoblotting analysis. **D**. The cells were treated with CuB and then stained with JC-1 probe before flow cytometry analysis. The loss of mitochondrial membrane potential was observed as a decrease in JC-1 red fluorescence (FL-2, 585 ± 40 nm) and an increase in JC-1 green fluorescence (FL-1, 530 ± 30 nm). **E**. The cytosolic fractions of control and cells treated with 1 μM CuB for 15 and 30 min were prepared and subjected to immunoprecipitation using an anti-cytochrome c antibody. **F**. The lysates of cells treated with 1 μM CuB for 6 h were prepared and subjected to immunoprecipitation using an anti-caspase-8 antibody.

Cofilin is a ubiquitous among eukaryotes and is responsible for the reorganization of actin filaments *in vivo*. Dephosphorylation of cofilin enables its actin severing and depolymerizing activity, and drives directional cell motility, thus providing a simple phospho-regulatory mechanism for actin reorganization [33]. Interestingly, once dephosphorylated, cofilin is translocated to mitochondria, where it contributes to mitochondrial membrane permeabilization, despolarization and the release of apoptotic factors, thus increasing apoptosis [22, 34]. Considering that previous studies have reported that CuB and CuE inhibit phosphorylation of cofilin [10, 16], we determined whether CuB-induced dephosphorylation of cofilin could lead it to translocate to mitochondria, resulting in mitochondrial dysfunction. We found that CuB induced dephosphorylation of cofilin rapidly and persistently (Figure 5A and 5B), precipitating translocation to mitochondria (Figure 5C) and thus loss of mitochondrial membrane potential, but no morphological effects were observed. The loss of mitochondrial membrane potential was observed as a decrease in JC-1 red fluorescence (FL-2, 585 ± 40 nm) and an increase in JC-1 green fluorescence (FL-1, 530 ± 30 nm). The control cells were fluoresced red, whereas CuB-treated cells turned green (Figure 5D).

In the mitochondrial-dependent apoptosis pathway, mitochondrial instability initiates apoptosome formation, and the sequential activation of caspase-8 and caspase-9 [35, 36]. We found that CuB induced the loss of mitochondrial membrane potential, resulting in activation of caspase-8 and release of cytochrome c (Figure 5F and 5E). Taken together, these results suggest that CuB-induced the mitochondrial-dependent apoptotic pathway, and was associated with persistent cofilin activation and its translocation to mitochondria.

### Anti–MM activity of CuB in xenograft mouse model

**Figure 6:**
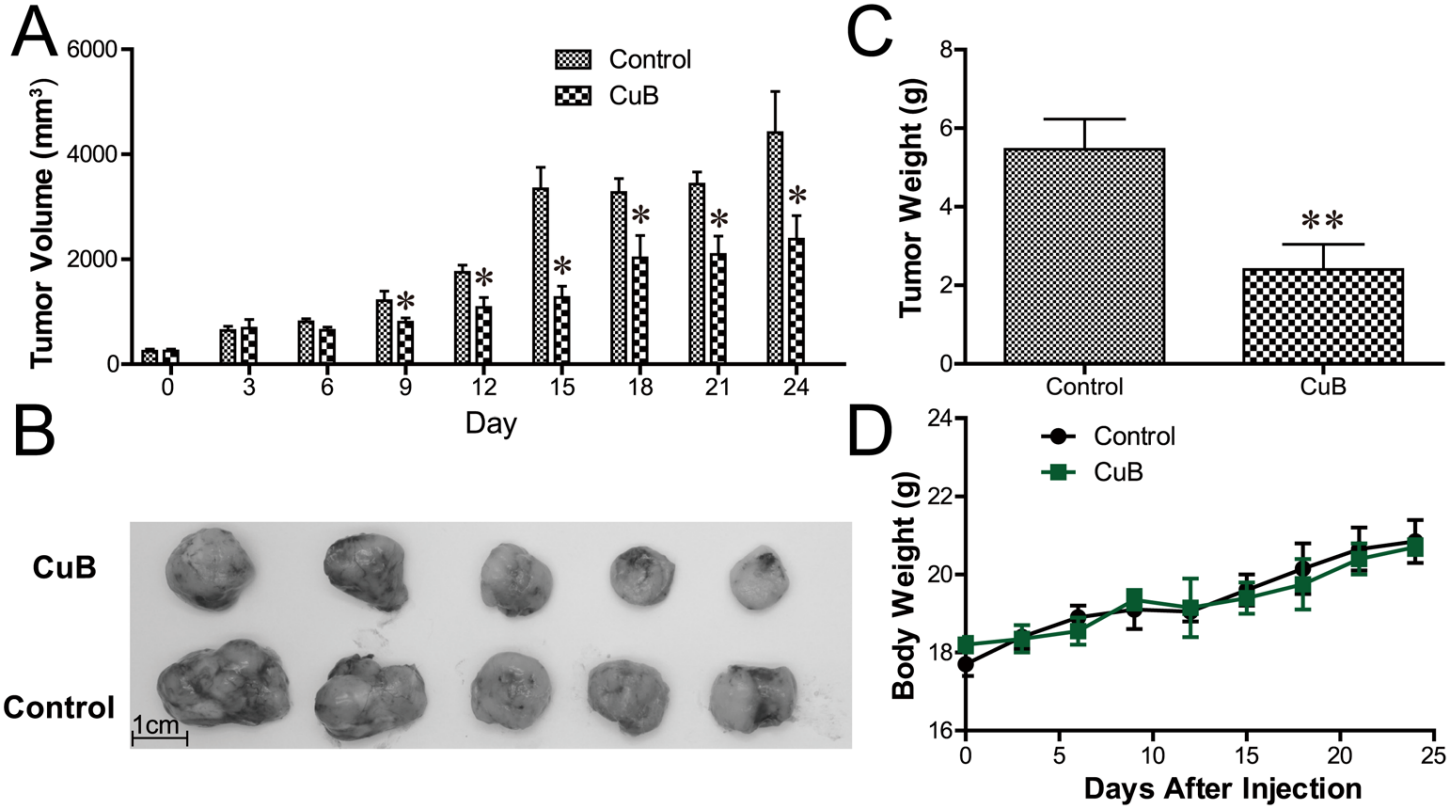
CuB inhibits growth of human MM xenograft tumors in SCID mice The CB17-SCID mice were inoculated subcutaneously with 3 × 10^7^ MM1.S cells. Intraperitoneal administration of CuB (0.1 mg/kg, three times per week) started after the development of a palpable tumor. The longest and shortest diameters of each tumor were measured with calipers at the indicated times. **A**. The tumor growth presented represents means ± SEM (n = 8). The volumes of CuB-treated animal tumors differed significantly from vehicle-treated controls (* *P* < 0.05). **B**. CuB significantly inhibited tumor growth and reduced tumor sizes. Representative tumors isolated from five drug-treated MM-bearing mice (CuB, upper panel) and five control mice (control, lower panel) are shown. **C**. The tumor weights were measured at the end of the *in vivo* assay ** *P* < 0.01. **D**. Body weights in CuB-treated animals did not significantly differ from vehicle-treated controls (*P* > 0.05).

We further examined the anti-MM activity of CuB *in vivo* using a xenograft model of human MM in severe combined immunodeficiency (SCID) mice. As shown in Figure 6A, CuB significantly inhibited tumor growth. After 24 days, the average tumor sizes were 4410.7 ± 1754.9 mm^3^, and 2381.0 ± 1005.3 mm^3^ in the control and CuB-treated groups, respectively (Figure 6B). The average tumor weights of the control group and CuB-treated group were 5.46 ± 2.18 g and 2.14 ± 1.27 g, respectively (Figure 6C). No significant differences in body weight were observed between CuB-treated and control animals (Figure 6D), suggesting that CuB causes low host toxicity at a therapeutic dose.

## DISCUSSION

This study was designed to demonstrate the anticancer effects of CuB, and to investigate the underlying mechanisms by which it may exert therapeutic activity in MM. Our results indicate that CuB significantly enhanced the therapeutic effects of Adriamycin in MM cells. Adriamycin has been used as a first-line treatment against relapsed/refractory MM [37]. However, a significant number of MM patients exhibit resistance to Adriamycin, and its cardiotoxicity and other side effects often require a combination regimen with other drugs to improve clinical condition and reduce host toxicity [38]. Accumulating evidence indicates that CuB potentiates the anti-proliferative effects of chemotherapy drugs in several human cancer cell lines [39], which makes CuB an attractive candidate with which a novel combination therapy could be developed. Our results demonstrated for the first time that combination of CuB and Adriamycin induced apoptosis and increased MM cell sensitivity to Adriamycin.

Secondly, we found that the anti-MM activity of CuB was dependent upon the *STAT3* pathway. The cytokine growth factor autocrine/paracrine loops play critical roles in hematopoietic growth and differentiation [40], resulting in abnormal, prolonged activation of cytokine-mediated transcription factors such as signal transducer and activators of transcription (*STAT*) in MM [41]. IL-10 growth factor is produced by the myeloma cells of about half of these patients, and induces abnormal continuous *STAT3* activation in malignant cells [42]. CuB was previously reported to block *STAT3* signaling by inhibiting its phosphorylation in various cancer cell lines [14, 43]. We tested the hypothesis that inhibitors of *STAT3* activation, such as CuB, could overcome this drug resistance and enhance the cytotoxic effects of chemotherapeutic drugs in cytokine-dependent MM tumor cells. In this study, RMPI8226, an MM cell line known to be dependent on interleukin IL-10 for survival, was used. Interestingly, IL-10 enhanced proliferation of RPMI8226 cells and inhibited Adriamycin-induced cell death. Further, we investigated whether CuB-induced inhibition of the IL-10/*STAT3* pathway could enhance the anti-proliferative effect of Adriamycin in RPMI8226 cells. The results indicated that the blockade of the *STAT3* pathway by CuB synergistically increased anti-tumor activity of Adriamycin. Taken together, these results reveal the molecular mechanism by which these two agents act in synergy to reduce growth of MM cells.

Thirdly, the role of CuB-induced inhibition of Aurora A protein kinase phosphorylation in its anti-MM activity was demonstrated. Protein kinase phosphorylation is one of the most common mechanisms regulating cell life and death, and abnormal phosphorylation can both cause or be a consequence of disease [44]. Orally active protein-kinase inhibitors have recently been approved for clinical use [45]. Many effective drugs act via modulation of multiple targets, rather single targets, which may be associated with superior efficacy and toxicity [46]. The target kinase of CuB has not yet been identified, so a high-throughput kinase screen was carried out. The specificities of CuB inhibition of a panel of up to 140 protein kinases was examined and indicated that 500 nM CuB inhibited the kinase activity of p-Aurora A by over 90%. Aurora kinases (AURKA, AURKB, and AURKC) are serine/threonine kinases that function as key regulators of mitosis [47]. Deregulation of Aurora kinases has been linked to tumorigenesis, and high Aurora A gene expression is correlated with centrosome amplification and proliferation in MM [48]. Thus, inhibitors of Aurora A may represent novel drug candidates for MM therapy. Expression of Aurora-A varies during the cell cycle, and is low during the G1 and S phases, peaking during the G2 and M phases, and dropping rapidly at the end of the M phase [49]. Down-regulation or depletion of the Aurora A gene is an essential component of G2-M arrest [50, 51], and Aurora inhibitors target diverse cellular processes and induce G2-M arrest [52, 53]. We found that CuB inhibited Aurora A and induced G2-M arrest and apoptosis of MM cells, indicating that CuB may more effectively inhibit cancer growth, especially in cancers with high levels of Aurora A expression.

Fourth, we elucidated the role of cofilin in CuB-Induced apoptosis. Cofilin is a member of the ADF/cofilin family, which regulates actin dynamics [54]. However, increasing evidence indicates that translocation of dephosphorylated cofilin to mitochondria is essential for regulation of apoptosis [22]. Interestingly, mitochondrial translocation of dephosphorylated cofilin was observed exclusively during apoptosis, whereas phosphorylation of cofilin suppressed mitochondrial translocation [23]. Although previous studies have reported that CuB induces cofilin dephosphorylation disrupts actin dynamics [10, 16], the relationship between CuB-induced dephosphorylation of cofilin and mitochondria remains unknown. We reported for the first time that CuB induces dephosphorylation of cofilin, which is translocated to mitochondria, causing mitochondrial dysfunction, release of cytochrome c, and activation of caspase-8. The results indicate that dephosphorylation of cofilin may contribute to CuB-mediated cytotoxicity in MM cells.

In this study, we measured Aurora phosphorylation using Western blotting. In this experiment, a short period of drug exposure (6 h) was used, so a higher dose of CuB was needed than in the proliferation assay. Aurora A auto-phosphorylation has been extensively investigated in various MM cell lines [48]. The effects of CuB on Aurora A auto-phosphorylation should be confirmed in other cell lines in the future. We demonstrated the effects of CuB on STAT3 phosphorylation in RPMI8226 cells. CuB was previously reported to block STAT3 signaling by inhibiting its phosphorylation in various cancer cell lines [14, 15]. This study focused on the role of IL-10 in MM cell line proliferation and resistance to anticancer drugs, considering that there are numerous reports on the role IL-6 in survival and proliferation of multiple myeloma (MM) cells through the phosphorylation of STAT3 [55]. Future studies should confirm whether similar results can be observed in other cell lines.

Our *in vitro* results indicated synergy between CuB and Adriamycin; and our *in vivo* data demonstrated that CuB effectively inhibited tumor growth in mice, without major host toxicity. However, we did not investigate the synergy between CuB and Adriamycin *in vivo* in this study. It should be noted that, demonstrating such synergy in the xenograft model *in vivo* would be more complicated and would require multiple concentrations of test compounds. In the future we hope to perform the required combination studies to investigate any drug-drug interactions, and establish whether the synergy observed *in vitro* occurs also in this mouse model.

In summary, our results reveal the effect of CuB on multiple pathways, and implicate these pathways in the mechanism by which CuB exerts anti-MM activity. CuB inhibited MM tumor growth in a murine MM model, without host toxicity, indicating that it might make a useful novel therapeutic agent for the treatment of MM. Future studies should investigate whether the mechanisms reported herein apply to other cancer types and in *in vivo* settings.

## MATERIALS AND METHODS

### Reagents, cells and cell culture

The multiple myeloma cell lines (MM1.S, MM1.R, RPMI8226, and U266) used in this study were obtained from the American Type Culture Collection (Rockville, MD, USA). The cells were grown in RPMI 1640 medium (Gibco, Gaithersburg, MD, USA) containing 10% (v/v) fetal bovine serum (FBS, GTBCO, Grand Island, NY, USA) and penicillin/streptomycin (Gibco). Cucurbitacins and Adriamycin were obtained from Sigma Aldrich (St. Louis, MO, USA) and dissolved each in dimethyl sulphoxide (DMSO) at a stock concentration of 10 mM (Sigma). The Aurora A kinase inhibitor MK-5108 and JAK1/2 inhibitor ruxolitinib were purchased from Selleck Chemicals (Houston, TX, USA). The drugs were dissolved in 100% DMSO to an initial stock solution of 10 mM, and aliquots were made and stored at -80°C until use.

### Cell viability assay

To explore whether CuB might have therapeutic potential against MM, proliferation assays were performed in dexamethasone-resistant (MM1.R), dexamethasone-sensitive (MM1.S), U266, and RPMI8226 cell lines, using the CCK-8 assay. The cytotoxicity assays were performed according to the protocol recommended by the Drug Evaluation Branch, National Cancer Institute, USA [56]. Briefly, cells were seeded in 96-well plates, at a density of 5 × 10^4^ per well. The cells were exposed to different concentrations of drugs for 48 h. Using Cell Counting Kit-8 for proliferation and cytotoxicity assays, the absorbance at 450 nm was measured using a microplate reader. Growth (%) was calculated for each drug at various concentration levels as follows: [(Ti-Tz)/(C-Tz)] × 100 for compound concentrations in which Ti ≥ Tz, or [(Ti-Tz)/Tz] × 100 for compound concentrations for which Ti ≤ Tz, where Tz is the OD at time 0, C is the OD of the control cells and TZ is the OD of test cells at various times (Ti)]. The drug concentration for growth inhibition of 50% (GI50) was also calculated for each drug.

### *In vitro* synergy testing

The effect of the combination of CuB with chemotherapeutic agent on proliferation was assessed by calculating combination index (CI) values using CalcuSyn software (Biosoft, Cambridge, UK). Based on the median-effect principle of Chou and Talalay [57], the CI provides a quantitative measure of the degree of interaction between multiple agents. The CI values of < 1, = 1 and > 1 denote as synergistic, additive and antagonistic effects, respectively. The fraction affected (Fa) was calculated from cell viability assays.

### Primary kinase screening

All protein kinases in the specificity panel were expressed, purified and assayed at The National Centre for Protein Kinase Profiling (http://www.kinase-screen.mrc.ac.uk/) as described previously [58]. To plates containing 0.5 μl of each, DMSO controls or acid blanks, 15 μl of kinase enzyme mix (defined in Figure 3) containing enzyme and peptide/protein substrate in buffer is added. Compounds are pre-incubated in the presence of the enzyme and peptide/protein substrate for 5 min before initiation of the reaction by addition of 10 μl of ATP (final concentration selected for each kinase at 500 nM). Assays are carried out for 30 min at room temperature (RT) before termination by the addition of 5 μl orthophosphoric acid. The assay plates are then harvested onto P81 Unifilter Plates by a Perkin Elmer Harvester and air dried. The dry Unifilter plates are then sealed by addition of MicroScint O and are counted in Perkin Elmer Topcount NXT scintillation counters. The results for each protein kinase tested were presented as remaining activity for each kinase.

### IC_50_ determination

The assays to determine the 50% inhibitory concentration (IC50) values for each enzyme were performed as described previously [59]. The substrate peptide (LRRLSLGLRRLSLGLRRLSLGLRRLSLG) of AurorA was used in kinase IC50 assays. In brief, Aurora A (5-20 mU diluted in 50 mM Tris pH=7.5, 0.1 mM EGTA, 0.1% β-mercaptoethanol, 1 mg/ml BSA) was assayed against LRRLSLGLRRLSLGLRRLSLGLRRLSLG in a final volume of 25.5 μL containing 50 mM Tris pH 7.5, 0.1 mM EGTA, 0.3 mM LRRLSLGLRRLSLGLRRLSLGLRRLSLG, 10 mM magnesium acetate, and 0.005 mM [33P-γ-ATP] (50-1000 cpm/pmole), and incubated for 30 min at RT. The assays are stopped by an addition of 5 μL of 0.5 M orthophosphoric acid and then harvested onto plates with a wash buffer of 50 mM orthophosphoric acid. The controls for included the solvents and the background phosphorylation levels were included in all assays and the non-specific ^32^P incorporation was subtracted from all the obtained values. IC50 values were generated from log dose-response curves using Prism 5 software (GraphPad Software, San Diego, CA, USA).

### Cell-cycle analysis and detection of apoptosis

The MM cells (5 × 10^5^) were cultured for 24 h in media alone or with CuB. Treated cells were fixed with 70% ethanol then 200 μg/ml RNase; the nuclei were stained with 50 μg/mL propidium iodide, and the cell cycle profile was assessed using an Accuri C6 flow cytometer (BD Biosciences, San Diego, CA, USA) using BD Accuri C6 software. Dual staining with FLUOS-labeled annexin V and PI was carried out to detect the apoptotic cells. After treatment of cells with CuB for 48 h, the cells were washed and double stained with Annexin V/ PI, using the Annexin-V-FLUOS staining kit (Roche, Basel, Switzerland). After incubation for 15 min at RT, the apoptotic cells were analyzed on a BD Accuri C6 Flow Cytometer.

### Preparation of mitochondrial and cytosolic fractions

The mitochondrial and cytosolic fractions were isolated using the mitochondria isolation kit (Pierce, Waltham, MA, USA) and protease inhibitor cocktail kit (Pierce, Waltham, MA, USA). Before starting the protein extraction, the protease inhibitor cocktail was added to the reagent according to the manufacturer's instruction. The MM1.S cell suspension was placed in a micro centrifuge tube and centrifuged at 850 g for 2 min; the supernatant was removed and discarded. To the resultant cell pellet, 800 μL of mitochondrial isolation reagent A was added; the mixture was then vortexed for 5 s and incubated on ice for 2 min. The mixture was transferred to Dounce Tissue Grinder (Sigma) and homogenized on ice for 30-50 passes. To the lysed cells, 800 μL of mitochondrial isolation reagent A was added and the mixture was vortexed for 5 s and then incubated on ice for 2 min; 10 μL of mitochondrial isolation reagent B was added and vortexed for 5 s. The mixture was incubated on ice for 5 min and vortexed at 21,000 g every one minute. Then, 800 μL of mitochondrial reagent C was added to this mixture and the tube was inverted several times to mix the reagents. This mixture was centrifuged at 700 g for 10 min at 4°C. The supernatant was transferred to a new tube and centrifuged at 3000 g for 15 min, in order to obtain more purified fraction of mitochondria with > 50% reduction in the lysosomal and peroxisomal contaminants. The supernatant containing the cytosolic fraction was carefully aspirated and transferred to a new tube and the pellet containing the mitochondria was re-suspended with 500 μL of reagent C and centrifuged at 12,000 g for 5 min. The supernatant was discarded and the pellet containing the mitochondria was dissolved in 100 μL of lysis buffer. The mixture was vortexed for 1 min and centrifuged at 21,000 g for 2 min and the supernatant containing the mitochondrial proteins was collected and used for protein quantification and Western blot analysis.

### Mitochondrial membrane potential assays

The cells (5 × 10^5^) were treated with various concentrations of CuB for 8 h and stained with the fluorescent probe JC-1 (mitochondria staining kit, Sigma) at 37°C for 20 min, according to the manufacturer's protocol. The mitochondrial membrane potential was quantified by measuring the fluorescence intensity emission shift from green (~529 nm) to red (~590 nm) using the BD Accuri C6 Flow Cytometer and CFlow software. Valinomycin, a known disruptor of the mitochondrial membrane potential, was used as a positive control.

### Western blotting analysis

The protein concentration of cell lysate samples was measured using the Bicinchoninic Acid Solution (BCA) protein assay kit (Thermo Fisher Scientific, San José, CA, USA). The cell lysates with equal amounts of total protein were mixed with an SDS loading buffer containing 10% SDS, 50 mM DTT, 312 mM Tris-HCl, and 50% glycerine, boiled for 5 min, and separated by SDS-PAGE electrophoresis. The proteins were then transferred to PVDF membranes (Roche Company, Basel, Switzerland) and the membranes were blocked with 5% nonfat dried milk in PBS containing 0.05% Tween 20, for 1 h at RT. The primary antibody incubations were performed in Tris-buffered saline with 0.1% Tween-20 (TTBS) with 5% nonfat dried milk overnight. After washing, horseradish peroxidase (HRP)-conjugated secondary antibodies, anti-rabbit IgG-HRP (Cell Signaling Technology, Danvers, MA, USA; 1:5000 dilution) and anti-mouse IgG-HRP (Cell Signaling Technology, Danvers, MA, USA; 1:5000 dilution) were added and incubated for 1 h. Subsequently, the membranes were washed and incubated in ECL Western blotting detection reagent (Millipore, Schwalbach, Germany). The following primary antibodies were used: rabbit anti-Phospho-Aurora (1:1000; Cell Signaling Technology, Danvers, MA, USA), rabbit anti-Aurora A (1:1000; Cell Signaling Technology), rabbit anti-Phospho-Histone H3 (1:1000; Cell Signaling Technology), rabbit anti-Phospho-p53 (1:1000; Cell Signaling Technology), rabbit anti-p53 (1:1000; Cell Signaling Technology), rabbit anti-Phospho-Cofilin (1:1000; Cell Signaling Technology), rabbit anti-Cofilin (1:1000; Cell Signaling Technology), rabbit anti-VDAC (1:1000; Cell Signaling Technology), rabbit anti-Cytochrome c (1:1000; Abcam, Cambridge, MA), rabbit anti-Caspase-8 (1:1000; Cell Signaling Technology), rabbit anti-Cleaved-Caspase-8 (1:1000; Cell Signaling Technology), and rabbit anti-*GAPDH* (1:1000; Cell Signaling Technology).

### Xenograft mouse models

All animal experiments were carried out according to the Chinese Laws of Animal Welfare and were reviewed and approved by the Ethics Commission of Chengdu Medical College, Chengdu, China. The female, 4 to 5 weeks old, CB17-SCID mice (Weitonglihua Company, Beijing, China) were housed under well-controlled conditions: 12 h:12 h light-dark cycle, 22 ± 1°C, 60 ± 10% humidity. Standard rodent diet and water were provided ad libitum. Animal body weight and any sign of morbidity were monitored throughout the experimental period. The mice were inoculated subcutaneously into their lower dorsum with 3 × 10^7^ MM1.S cells in 100 μL of PBS and 100 μL of Matrigel (Corning, NY, USA). When tumors were palpable, the mice were randomly assigned to receive intraperitoneal (i.p.) injection of CuB (0.1 mg/kg, 100 μL, three times per week for up to 4 weeks) or vehicle (PBS, 100 μL). The shortest and longest diameters of the tumors were measured with electronic calipers at the indicated time intervals, and the tumor volumes were calculated using following formula: (the longest diameter) × (the shortest diameter)^2^ × 0.5. When average tumor volume reached 2 cm^3^, mice were sacrificed, according to institutional guidelines. At the end of the experiment, the tumors were extirpated, measured and weighed.

### Statistical analysis

All the *in vitro* experiments were repeated at least three times and the data were expressed as means ± standard deviations (SDs). Statistical significances of the differences were determined by Student's *t*-test. *P* value < 0.05 was considered statistically significant.

## References

[1] Mahindra A, Laubach J, Raje N, Munshi N, Richardson PG, Anderson K (2012). Latest advances and current challenges in the treatment of multiple myeloma. Nat Rev Clin Oncol.

[2] Raab MS, Podar K, Breitkreutz I, Richardson PG, Anderson KC (2009). Multiple myeloma. Lancet.

[3] Sandru A, Voinea S, Panaitescu E, Blidaru A (2014). Survival rates of patients with metastatic malignant melanoma. Journal of medicine and life.

[4] Cragg GM, Grothaus PG, Newman DJ (2014). New horizons for old drugs and drug leads. J Nat Prod.

[5] Han T, Ma H, Chao Y, Chou L, Chung-Hua I (1979). Preventive effects of cucurbitacin B on experimental hepatitis and cirrhosis. Chung-hua I Hsueh Tsa Chih (Beijing).

[6] Sun Y, Zhang J, Zhou J, Huang Z, Hu H, Qiao M, Zhao X, Chen D (2015). Synergistic effect of cucurbitacin B in combination with curcumin via Enhancing apoptosis induction and reversing multidrug resistance in Human hepatoma cells. European journal of pharmacology.

[7] Song J, Liu H, Li Z, Yang C, Wang C (2015). Cucurbitacin I inhibits cell migration and invasion and enhances chemosensitivity in colon cancer. Oncology reports.

[8] Gupta P, Srivastava SK (2014). Inhibition of Integrin-HER2 signaling by Cucurbitacin B leads to in vitro and in vivo breast tumor growth suppression. Oncotarget.

[9] Zheng Q, Liu Y, Liu W, Ma F, Zhou Y, Chen M, Chang J, Wang Y, Yang G, He G (2014). Cucurbitacin B inhibits growth and induces apoptosis through the JAK2/STAT3 and MAPK pathways in SHSY5Y human neuroblastoma cells. Molecular medicine reports.

[10] Zhu JS, Ouyang DY, Shi ZJ, Xu LH, Zhang YT, He XH (2012). Cucurbitacin B induces cell cycle arrest, apoptosis and autophagy associated with G actin reduction and persistent activation of cofilin in Jurkat cells. Pharmacology.

[11] Iwanski GB, Lee DH, En-Gal S, Doan NB, Castor B, Vogt M, Toh M, Bokemeyer C, Said JW, Thoennissen NH, Koeffler HP, Cucurbitacin B (2010). a novel in vivo potentiator of gemcitabine with low toxicity in the treatment of pancreatic cancer. British journal of pharmacology.

[12] Shukla S, Khan S, Kumar S, Sinha S, Farhan M, Bora HK, Maurya R, Meeran SM (2015). Cucurbitacin B Alters the Expression of Tumor-Related Genes by Epigenetic Modifications in NSCLC and Inhibits NNK-Induced Lung Tumorigenesis. Cancer prevention research.

[13] Guo J, Wang J, Cai C, Xu J, Yu H, Xu H, Xing T (2015). The anti-melanoma efficiency of the intratumoral injection of cucurbitacin-loaded sustained release carriers: in situ-forming implants. AAPS PharmSciTech.

[14] Chan KT, Li K, Liu SL, Chu KH, Toh M, Xie WD (2010). Cucurbitacin B inhibits STAT3 and the Raf/MEK/ERK pathway in leukemia cell line K562. Cancer letters.

[15] Dong Y, Lu B, Zhang X, Zhang J, Lai L, Li D, Wu Y, Song Y, Luo J, Pang X, Yi Z, Liu M, Cucurbitacin E (2010). a tetracyclic triterpenes compound from Chinese medicine, inhibits tumor angiogenesis through VEGFR2-mediated Jak2-STAT3 signaling pathway. Carcinogenesis.

[16] Nakashima S, Matsuda H, Kurume A, Oda Y, Nakamura S, Yamashita M, Yoshikawa M (2010). Cucurbitacin E as a new inhibitor of cofilin phosphorylation in human leukemia U937 cells. Bioorg Med Chem Lett.

[17] Ren G, Sha T, Guo J, Li W, Lu J, Chen X (2015). Cucurbitacin B induces DNA damage and autophagy mediated by reactive oxygen species (ROS) in MCF-7 breast cancer cells. Journal of natural medicines.

[18] Amin HM, McDonnell TJ, Ma Y, Lin Q, Fujio Y, Kunisada K, Leventaki V, Das P, Rassidakis GZ, Cutler C (2004). Selective inhibition of STAT3 induces apoptosis and G1 cell cycle arrest in ALK-positive anaplastic large cell lymphoma. Oncogene.

[19] Sun X, Zhang J, Wang L, Tian Z (2008). Growth inhibition of human hepatocellular carcinoma cells by blocking STAT3 activation with decoy-ODN. Cancer letters.

[20] Premkumar DR, Jane EP, Pollack IF (2015). Cucurbitacin-I inhibits Aurora kinase A, Aurora kinase B and survivin, induces defects in cell cycle progression and promotes ABT-737-induced cell death in a caspase-independent manner in malignant human glioma cells. Cancer biology & therapy.

[21] Ma H, Horiuchi KY (2006). Chemical microarray: a new tool for drug screening and discovery. Drug discovery today.

[22] Li GB, Cheng Q, Liu L, Zhou T, Shan CY, Hu XY, Zhou J, Liu EH, Li P, Gao N (2013). Mitochondrial translocation of cofilin is required for allyl isothiocyanate-mediated cell death via ROCK1/PTEN/PI3K signaling pathway. Cell communication and signaling.

[23] Chua BT, Volbracht C, Tan KO, Li R, Yu VC, Li P (2003). Mitochondrial translocation of cofilin is an early step in apoptosis induction. Nature cell biology.

[24] Zhou N, Singh K, Mir MC, Parker Y, Lindner D, Dreicer R, Ecsedy JA, Zhang Z, Teh BT, Almasan A, Hansel DE (2013). The investigational Aurora kinase A inhibitor MLN8237 induces defects in cell viability and cell-cycle progression in malignant bladder cancer cells in vitro and in vivo. Clinical cancer research.

[25] Shimomura T, Hasako S, Nakatsuru Y, Mita T, Ichikawa K, Kodera T, Sakai T, Nambu T, Miyamoto M, Takahashi I, Miki S, Kawanishi N, Ohkubo M, Kotani H, Iwasawa Y (2010). MK-5108, a highly selective Aurora-A kinase inhibitor, shows antitumor activity alone and in combination with docetaxel. Mol Cancer Ther.

[26] Munshi A, Ramesh R (2013). Mitogen-activated protein kinases and their role in radiation response. Genes Cancer.

[27] Liu X, Xiao W, Wang XD, Li YF, Han J, Li Y (2013). The p38-interacting protein (p38IP) regulates G2/M progression by promoting alpha-tubulin acetylation via inhibiting ubiquitination-induced degradation of the acetyltransferase GCN5. The Journal of biological chemistry.

[28] Vogt J, Traynor R, Sapkota GP (2011). The specificities of small molecule inhibitors of the TGFss and BMP pathways. Cellular signalling.

[29] Tomita M, Aurora Mori N (2010). A selective inhibitor MLN8237 suppresses the growth and survival of HTLV-1-infected T-cells in vitro. Cancer science.

[30] Katayama H, Sasai K, Kawai H, Yuan ZM, Bondaruk J, Suzuki F, Fujii S, Arlinghaus RB, Czerniak BA, Sen S (2004). Phosphorylation by aurora kinase A induces Mdm2-mediated destabilization and inhibition of p53. Nature genetics.

[31] Zhang M, Bian ZG, Zhang Y, Wang JH, Kan L, Wang X, Niu HY, He P (2014). Cucurbitacin B inhibits proliferation and induces apoptosis via STAT3 pathway inhibition in A549 lung cancer cells. Molecular medicine reports.

[32] Kovacs E (2010). Interleukin-6 leads to interleukin-10 production in several human multiple myeloma cell lines. Does interleukin-10 enhance the proliferation of these cells?. Leuk Res.

[33] Huang TY, DerMardirossian C, Bokoch GM (2006). Cofilin phosphatases and regulation of actin dynamics. Current opinion in cell biology.

[34] Posadas I, Perez-Martinez FC, Guerra J, Sanchez-Verdu P, Cena V (2012). Cofilin activation mediates Bax translocation to mitochondria during excitotoxic neuronal death. Journal of neurochemistry.

[35] Cowling V, Downward J (2002). Caspase-6 is the direct activator of caspase-8 in the cytochrome c-induced apoptosis pathway: absolute requirement for removal of caspase-6 prodomain. Cell death and differentiation.

[36] Chen M, Guerrero AD, Huang L, Shabier Z, Pan M, Tan TH, Wang J (2007). Caspase-9-induced mitochondrial disruption through cleavage of anti-apoptotic BCL-2 family members. The Journal of biological chemistry.

[37] Samson D, Gaminara E, Newland A, Van de Pette J, Kearney J, McCarthy D, Joyner M, Aston L, Mitchell T, Hamon M (1989). Infusion of vincristine and doxorubicin with oral dexamethasone as first-line therapy for multiple myeloma. Lancet.

[38] Octavia Y, Tocchetti CG, Gabrielson KL, Janssens S, Crijns HJ, Moens AL (2012). Doxorubicin-induced cardiomyopathy: from molecular mechanisms to therapeutic strategies. Journal of molecular and cellular cardiology.

[39] Lee DH, Thoennissen NH, Goff C, Iwanski GB, Forscher C, Doan NB, Said JW, Phillip Koeffler H (2011). Synergistic effect of low-dose cucurbitacin B and low-dose methotrexate for treatment of human osteosarcoma. Cancer letters.

[40] Robb L (2007). Cytokine receptors and hematopoietic differentiation. Oncogene.

[41] Alas S, Bonavida B (2003). Inhibition of constitutive STAT3 activity sensitizes resistant non-Hodgkin's lymphoma and multiple myeloma to chemotherapeutic drug-mediated apoptosis. Clinical cancer research.

[42] Klein B, Lu ZY, Gu ZJ, Costes V, Jourdan M, Rossi JF (1999). Interleukin-10 and Gp130 cytokines in human multiple myeloma. Leukemia & lymphoma.

[43] Liu T, Peng H, Zhang M, Deng Y, Wu Z, Cucurbitacin B (2010). a small molecule inhibitor of the Stat3 signaling pathway, enhances the chemosensitivity of laryngeal squamous cell carcinoma cells to cisplatin. European journal of pharmacology.

[44] Suo SB, Qiu JD, Shi SP, Chen X, Liang RP, PSEA (2014). Kinase-specific prediction and analysis of human phosphorylation substrates. Scientific reports.

[45] Cohen P (2002). Protein kinases—the major drug targets of the twenty-first century?. Nature reviews Drug discovery.

[46] Hopkins AL (2008). Network pharmacology: the next paradigm in drug discovery. Nature chemical biology.

[47] Marumoto T, Honda S, Hara T, Nitta M, Hirota T, Kohmura E, Saya H (2003). Aurora-A kinase maintains the fidelity of early and late mitotic events in HeLa cells. The Journal of biological chemistry.

[48] Gorgun G, Calabrese E, Hideshima T, Ecsedy J, Perrone G, Mani M, Ikeda H, Bianchi G, Hu Y, Cirstea D, Santo L, Tai YT, Nahar S, Zheng M, Bandi M, Carrasco RD (2010). A novel Aurora-A kinase inhibitor MLN8237 induces cytotoxicity and cell-cycle arrest in multiple myeloma. Blood.

[49] Zhu X, Mei J, Wang Z (2014). Aurora-A kinase: potential tumor marker of osteosarcoma. Journal of cancer research and therapeutics.

[50] Lee SY, Lee GR, Woo DH, Park NH, Cha HJ, Moon YH, Han IS (2013). Depletion of Aurora A leads to upregulation of FoxO1 to induce cell cycle arrest in hepatocellular carcinoma cells. Cell cycle.

[51] Lu Y, Liu Y, Jiang J, Xi Z, Zhong N, Shi S, Wang J, Wei X (2014). Knocking down the expression of Aurora-A gene inhibits cell proliferation and induces G2/M phase arrest in human small cell lung cancer cells. Oncology reports.

[52] Li JP, Yang YX, Liu QL, Pan ST, He ZX, Zhang X, Yang T, Chen XW, Wang D, Qiu JX, Zhou SF (2015). The investigational Aurora kinase A inhibitor alisertib (MLN8237) induces cell cycle G2/M arrest, apoptosis, and autophagy via p38 MAPK and Akt/mTOR signaling pathways in human breast cancer cells. Drug design, development and therapy.

[53] Cha TL, Chuang MJ, Wu ST, Sun GH, Chang SY, Yu DS, Huang SM, Huan SK, Cheng TC, Chen TT, Fan PL, Hsiao PW (2009). Dual degradation of aurora A and B kinases by the histone deacetylase inhibitor LBH589 induces G2-M arrest and apoptosis of renal cancer cells. Clinical cancer research.

[54] Blanchoin L, Boujemaa-Paterski R, Sykes C, Plastino J (2014). Actin dynamics, architecture, and mechanics in cell motility. Physiological reviews.

[55] Bharti AC, Donato N, Aggarwal BB (2003). Curcumin (diferuloylmethane) inhibits constitutive and IL-6-inducible STAT3 phosphorylation in human multiple myeloma cells. Journal of immunology.

[56] Karali N (2002). Synthesis and primary cytotoxicity evaluation of new 5-nitroindole-2,3-dione derivatives. Eur J Med Chem.

[57] Chou TC, Talalay P (1984). Quantitative analysis of dose-effect relationships: the combined effects of multiple drugs or enzyme inhibitors. Advances in enzyme regulation.

[58] Pearce LR, Alton GR, Richter DT, Kath JC, Lingardo L, Chapman J, Hwang C, Alessi DR (2010). Characterization of PF-4708671, a novel and highly specific inhibitor of p70 ribosomal S6 kinase (S6K1). Biochem J.

[59] Tyler RK, Shpiro N, Marquez R, Eyers PA (2007). VX-680 inhibits Aurora A and Aurora B kinase activity in human cells. Cell cycle.

